# A Bibliometric Analysis of Alternate-Day Fasting from 2000 to 2023

**DOI:** 10.3390/nu15173724

**Published:** 2023-08-25

**Authors:** Xiaoxiao Lin, Shuai Wang, Jinyu Huang

**Affiliations:** Affiliated Hangzhou First People’s Hospital, Zhejiang University School of Medicine, Hangzhou 310030, China; linxiaoxiao@zcmu.edu.cn

**Keywords:** alternate-day fasting, bibliometric analysis, weight loss, obesity, cardiometabolic risk

## Abstract

Alternate-day fasting (ADF) is becoming more popular since it may be a promising diet intervention for human health. Our study aimed to conduct a comprehensive bibliometric analysis to investigate current publication trends and hotspots in the field of ADF. Publications regarding ADF were identified from the Web of Science Core Collection (WOSCC) database. VOSviewer 1.6.16 and Online Analysis Platform were used to analyze current publication trends and hotspots. In total, there were 184 publications from 362 institutions and 39 countries/regions, which were published in 104 journals. The most productive countries/regions, institutions, authors, and journals were the USA, University of Illinois Chicago, Krista A. Varady, and *Nutrients*, respectively. The first high-cited publication was published in *PNAS* and authored by R. Michael Anson, and it was also the first article about ADF. The top five keywords with the highest frequency were as follows: calorie restriction, weight loss, intermittent fasting, obesity, and body weight. In conclusion, this is the first comprehensive bibliometric analysis related to ADF. The main research hotspots and frontiers are ADF for obesity and cardiometabolic risk, and ADF for several different population groups including healthy adults and patients with diabetes, nonalcoholic fatty liver disease (NAFLD), and cancer. The number of studies about ADF is relatively small, and more studies are needed to extend our knowledge about ADF, to improve human health.

## 1. Introduction

Alternate-day fasting (ADF), which is defined as a feast day with usual food, alternated with a fast day with a calorie restriction of about 25% of usual intake (approximately 500 kcal), is a main type of intermittent fasting (IF) [1,2,3,4,5,6]. ADF is usually used for people with obesity and overweight. A previous systematic review and meta-analysis demonstrated that ADF could effectively lower body fat mass (FM), body weight (BW), total cholesterol (TC), and body mass index (BMI) in individuals with obesity [7]. In addition to obesity, ADF can be used to manage other diseases including non-alcoholic fatty liver disease (NAFLD), diabetes, and asthma. For example, a recent study showed that the combination of exercise with ADF was effective for reducing hepatic steatosis in individuals with NAFLD [8]. In addition, ADF is also beneficial to healthy adults, and Slaven Stekovic et al. found that ADF could reduce low-density lipoprotein, the level of sICAM-1 (an age-associated inflammatory marker), and the metabolic regulator triiodothyronine in healthy and non-obese humans [9]. It seems that ADF could become a beneficial intervention for diverse population groups.

Recently, ADF is becoming more popular since it may be a promising diet intervention for human health. Despite the growing popularity of ADF, there is no bibliometric study summarizing the current publication trends and predicting research hotspots in this field.

Bibliometric study is a comprehensive and timely analysis of countries/regions, institutions, authors, keywords, h-index, and other parameters related to all publication in a specific field. It can provide a detailed overview of a specific area of knowledge and help the researchers know the current publication trends and find hotspots. VOSviewer is a popular software tool used for bibliometric analysis, and the key functions of VOSviewer include the following: (1) network visualization, which allows users to create various types of bibliometric networks, such as co-authorship networks, co-citation networks, and keyword co-occurrence networks; (2) clustering and mapping, which employs advanced algorithms to cluster nodes (authors, articles, or keywords) that are closely related within the network; (3) keyword analysis, which can reveal trends and emerging topics in a particular research area by identifying commonly used terms and their relationships; (4) citation analysis, which helps users explore citation patterns among research articles. This can provide insights into influential articles, research trends, and the evolution of scientific ideas over time. Therefore, our study aimed to conduct a comprehensive bibliometric analysis to determine the frontiers and hotspots in the field of ADF, and then provide a panoramic vision and guidance for future researchers.

## 2. Materials and Methods

### 2.1. Search Strategy

In our study, the relevant documents were extracted from Web of Science Core Collection (WoSCC). The database was queried using the following terms: “alternate-day fasting” or “modified alternate day fasting” or “alternate day fasting” or “modified alternate-day fasting” or “alternate day calorie restriction” or “alternate-day calorie restriction” or “modified alternate-day calorie restriction” or “alternate-day modified fasting” from 1 January 2000 to 30 May 2023, with articles and reviews, in the English language.

### 2.2. Data Collection and Bibliometric Analysis

After the selection, the document in TXT format with “Full Record and Cited References” was downloaded and imported in the VOSviewer 1.6.16 software. We performed a two-step analysis by WoSCC Online Analysis Platform and VOSviewer 1.6.16 software. The information about annual publication number, the top 10 productive countries/regions, institutions, authors, and journals, and the top 20 high-cited publications were exported from WoSCC Online Analysis Platform. VOSviewer 1.6.16 was used to analyze the co-authorship of institutions, countries/regions, authors, citation of journals and references, co-citation of references, and co-occurrence of keywords, and then output relevant figures. In the keyword co-occurrence analysis, we merged the synonyms of “alternate-day fasting” or “modified alternate day fasting” or “alternate day fasting” or “modified alternate-day fasting” or “alternate day calorie restriction” or “alternate-day calorie restriction” or “modified alternate-day calorie restriction” or “alternate-day modified fasting” into the term “alternate-day fasting”, “caloric restriction” and “calorie restriction” into the term “caloric restriction”, “weight loss” and “weight-loss” into the term “weight loss”, “body-composition” and “body composition” into the term “body composition”, and “insulin resistance” and “insulin-resistance” into the term “insulin resistance”.

## 3. Results

### 3.1. Quantity and Trends Analysis of Published Papers

**Figure 1:**
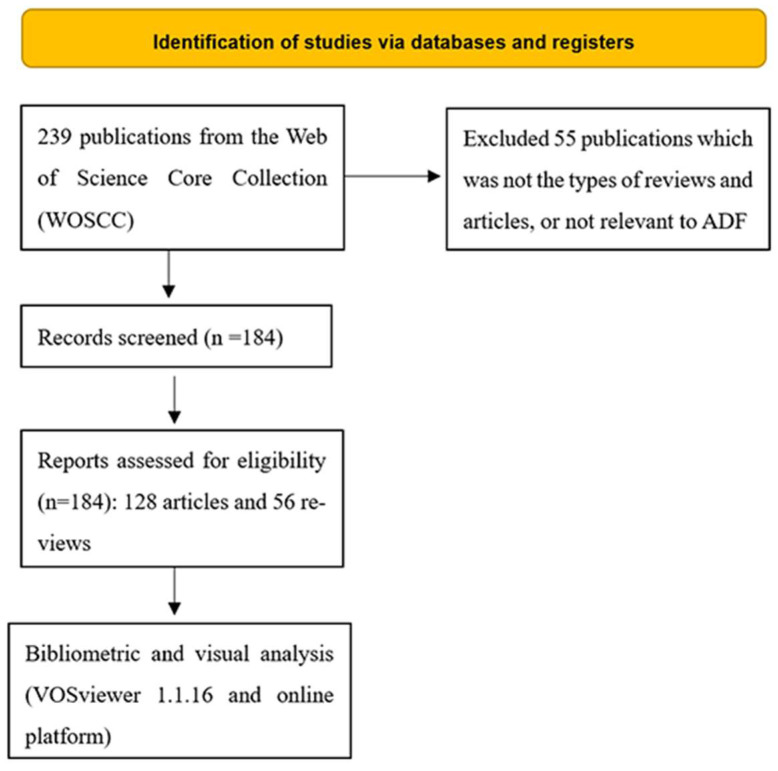
Flowchart of the inclusion and exclusion criteria.

**Figure 2:**
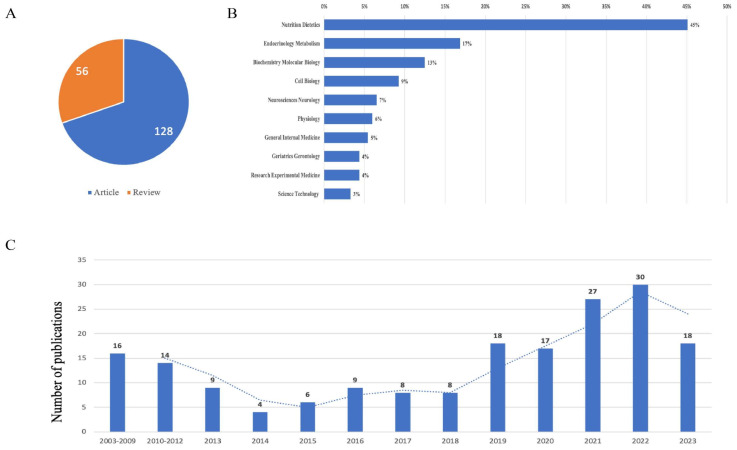
Yearly quantity and literature type of publications on ADF from inception to 30 May 2023. (**A**) Literature type distribution. (**B**) Subject category distribution. (**C**) Annual publication quantitative distribution.

In general, 184 publications including 128 articles and 56 reviews met the inclusion criteria, as shown in Figure 1. The publications, the types of publications, and the subject categories are listed in Figure 2. The top two subjects were nutrition dietetics with 83 publications and endocrinology metabolism with 31 publications. The time periods of publications could be divided into three phases: the first phase included documents published before 2010 (2003–2009), with about 16 documents published on ADF; the second phase included documents published between 2010 and 2018, with no significant increase in the number of publications in the field of ADF; in 2019, the number of publications showed a sharp upward trend, representing the third phase, from 2019 until now.

### 3.2. Analysis of Countries/Regions, Institutions, and Authors

**Figure 3:**
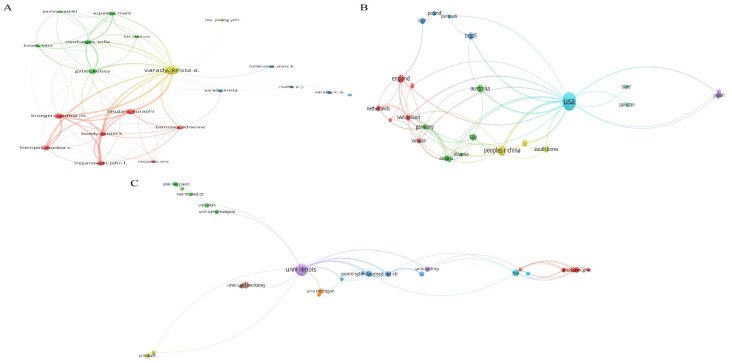
Visualization knowledge maps of authors, institutions, and countries/regions. (**A**) The co-authorship map of authors. (**B**) The co-authorship map of countries/regions. (**C**) The co-authorship map of institutions. Different colors indicate different clusters, and the node size indicates the number of publications. The thickness of the lines represents the link strength of the authors, countries/regions, and institutions.

**Figure 4:**
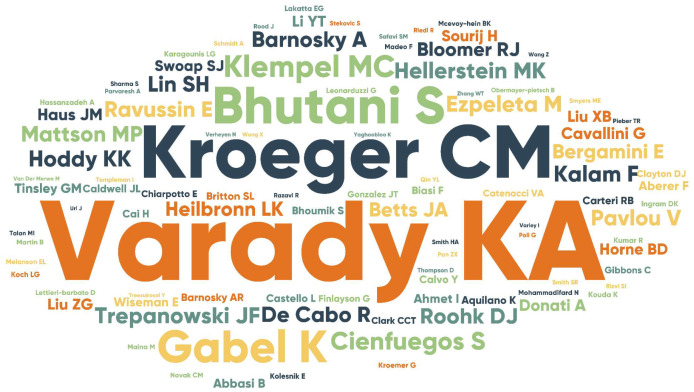
Word cloud of frequent authors’ keywords. A word cloud is a visual representation of words, giving greater prominence to words that appear more frequently.

In total, 362 institutions from 39 countries/regions contributed to the field of ADF. The most productive institutions were the University of Illinois Chicago with 37 publications and 2212 citations, the National Institutes of Health (NIH) with 10 publications and 1439 citations, and the University of California System with 9 publications and 490 citations. More than half of total publications were from the USA, accounting for 53.3% (98/184), followed by China with 23 publications, and England with 14 publications. In this field, Krista A. Varady was the most productive author with 43 publications, which is far more than others; she had a high h-index of 25 and a total of 2628 citations. After her, Cynthia M. Kroeger with 17 publications and Monica C. Klempel with 13 publications were the second and third most productive authors. The top 10 most active authors, institutions, and countries/regions in the field of ADF are summarized in Table 1. The network visualization maps of the cooperation relation among authors, organizations, and countries were shown in Figure 3. The top three cooperative authors were Krista A. Varady, Cynthia M Kroeger, and Kelsey Gabel. The top three cooperative institutions were the University of Illinois Chicago, the Medical University of Graz, and the National Institutes of Health (NIH). The top three cooperative countries were the USA, Switzerland, and Germany. The word cloud representing the most frequent authors’ keywords is displayed in Figure 4.

### 3.3. Analysis of Journals and Highly Cited Publications

**Figure 5:**
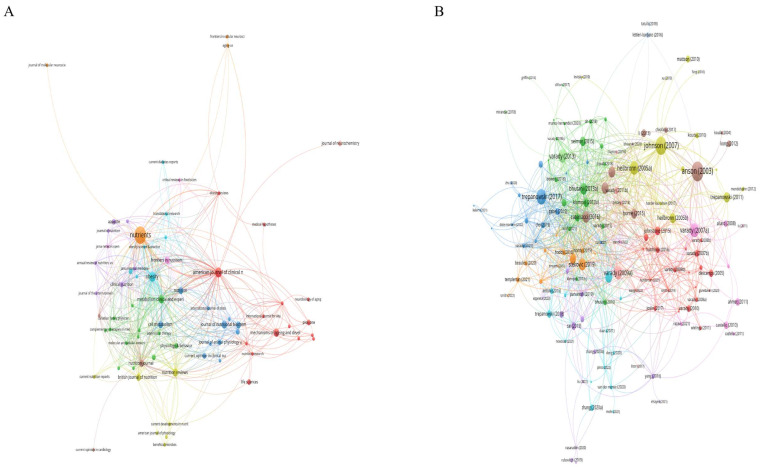
Visualization knowledge maps of journals and references. (**A**) Citation of journals. (**B**) Citation of references. The top three most productive journals were *Nutrients, American Journal of Clinical Nutrition*, and *Obesity.* The top three most highly cited publications were “Intermittent fasting dissociates beneficial effects of dietary restriction on glucose metabolism and neuronal resistance to injury from calorie intake” [10], “Alternate-day calorie restriction improves clinical findings and reduces markers of oxidative stress and inflammation in overweight adults with moderate asthma” [17], and “A randomized pilot study comparing zero-calorie alternate-day fasting to daily caloric restriction in adults with obesity” [13].

Over the last 20 years, 184 documents were published in 104 journals. *Nutrients* was the most productive journal with 18 publications. After it, *American Journal of Clinical Nutrition* and *Obesity* both had six publications. The top 10 productive journals in the field of ADF are shown in Table 2. The characteristics of the top 20 high-cited publications [9,10,11,12,13,14,15,16,17,18,19,20,21,22,23,24,25,26,27,28] are summarized in Table 3, and the most highly cited publication was published in *PNAS* and authored by R. Michael Anson et al., in 2003 [10]. It was also the first article about ADF. In this article, they found that intermittent fasting with ADF had more benefits including increased resistance of neurons to excitotoxic stress in the brain and reduced insulin levels and serum glucose compared with caloric restriction in mice. The second most highly cited publication was published in *Free Radical Biology and Medicine*, authored by James B. Johnson et al., in 2007 [17]. In this article, they found that ADF could decrease the indicators of inflammation, including brain-derived neurotrophic factor and serum tumor necrosis factor-α (TNF-α). The third most highly cited publication was published in *JAMA Internal Medicine* in 2017, authored by Trepanowski et al. [24]. In this article, 69 metabolically healthy obese adults were included. Compared with a daily calorie restriction diet, ADF was not superior in some factors including weight maintenance, weight loss, adherence, and improvement in risk indicators for cardiovascular disease (CVD). The fourth most highly cited publication was published in *American Journal of Clinical Nutrition* in 2007, authored by Dr. Krista A. Varady and Dr. Mark K Hellerstein. In this review, they summarized the human and animal trials in ADF and the prevention of chronic diseases including type 2 diabetes (T2DM) and cardiovascular disease (CVD), since ADF may modulate several risk factors effectively and prevent these chronic diseases to a similar extent to CR. The network visualization maps of citations of journals and references are shown in Figure 5.

### 3.4. Analysis of Document Co-Citation and Clustered Network

**Figure 6:**
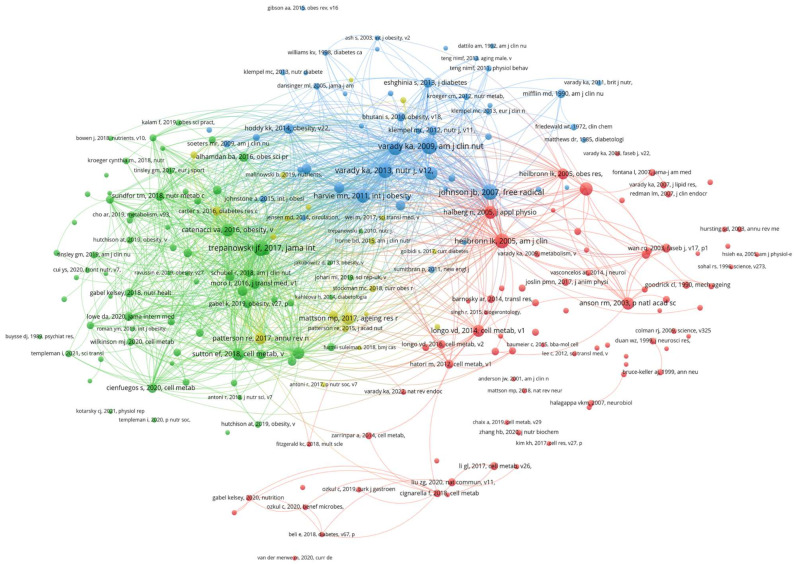
The network visualization map of co-citation of references. The top four co-cited references were “Alternate-day fasting and chronic disease prevention: A review of human and animal trials” [28] (54 co-citations), “Effect of alternate-day fasting on weight loss, weight maintenance, and cardioprotection among metabolically healthy obese adults: A randomized clinical trial” [24] (51 co-citations), “Alternate-day calorie restriction improves clinical findings and reduces markers of oxidative stress and inflammation in overweight adults with moderate asthma” (50 co-citations), and “Alternate-day fasting for weight loss in normal weight and overweight subjects: A randomized controlled trial” [27] (47 co-citations).

Figure 6 shows the co-citation reference in the field of ADF. Co-cited references are defined where one publication is cited by more than one article of the 184 extracted list. The top three co-cited references were Dr. Krista A Varady et al., American journal of clinical nutrition, in 2007 (54 co-citations), Trepanowski et al., *JAMA Internal Medicine*, in 2017 (51 co-citations), and James B. Johnson et al., *Free Radical Biology and Medicine*, in 2007 (50 co-citations), which were described above. The fourth-ranked co-cited reference was published in *Nutrition Journal* with 47 co-citations, authored by Dr. Krista A Varady et al., in 2013. In this randomized controlled trial, they found that ADF was effective for cardioprotection and weight loss in adults with and without overweight. The network visualization map of the co-citation of references is shown in Figure 6.

### 3.5. Analysis of Keywords

**Figure 7:**
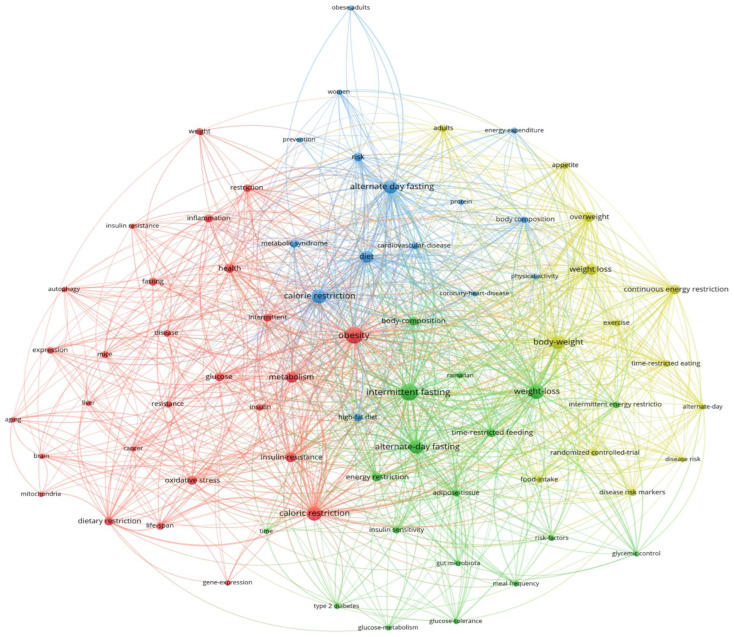
Visualization of keyword co-occurrence analysis. Four clusters are displayed. The top 10 core keywords aside from alternate-day fasting were calorie restriction (N = 81), weight loss (N = 70), intermittent fasting (N = 62), obesity (N = 62), body weight (N = 41), diet (N = 32), insulin resistance (N = 29), metabolism (N = 28), overweight (N = 23), and body composition (N = 22).

The co-occurrence of keywords, which could be classified in four clusters, is shown in Figure 7, presenting the frontiers, trends, and hot topics in this field. The green cluster includes alternate-day fasting, weight loss, body weight, and overweight. The red cluster includes caloric restriction, obesity, and metabolism. The blue cluster includes insulin resistance and energy restriction. The yellow cluster includes intermittent fasting, diet, and body composition. The top 10 keywords with the highest frequency aside form alternate-day fasting were calorie restriction (N = 81), weight loss (N = 70), intermittent fasting (N = 62), obesity (N = 62), body weight (N = 41), diet (N = 32), insulin resistance (N = 29), metabolism (N = 28), overweight (N = 23), and body composition (N = 22).

## 4. Discussion

### 4.1. General Information

To the best of knowledge, this is the first bibliometric analysis related to ADF. ADF is a main type of intermittent fasting, with many benefits for human health, including weight loss and improvements in cardiometabolic parameters and glucose regulation; many previous studies explored the effects of ADF for health conditions including obesity, NAFLD, and diabetes [29,30]. In our analysis, there were 184 publications of 127 articles and 57 reviews from 362 institutions and 39 countries/regions, which were published in 104 journals about ADF. The most productive countries/regions, institutions, authors, and journals were the USA with 98 publications, the University of Illinois Chicago with 37 publications, Krista A. Varady with 43 publications, and Nutrients with 18 publications, respectively. The most highly cited publication was published in PNAS, authored by R. Michael Anson et al., in 2003 [10], and it was also the first article about ADF. The top five keywords with the highest frequency were as follows: calorie restriction, weight loss, intermittent fasting, obesity, and body weight.

### 4.2. Hotspots and Frontiers

On the basis of current publication trends, important keywords with high frequency, and highly cited publications, the research hotspots in the field of ADF were summarized as follows: (1) **ADF for obesity and cardiometabolic risk.** In the top 20 highly cited references, seven explored the effects of ADF for obesity, and metabolic and cardiometabolic risk [12,13,19,22,24,26,27]. For core keywords, weight loss, body weight, and overweight occurred in the green cluster, while obesity and metabolism were in the red cluster. A meta-analysis demonstrated that ADF could lower body weight, body mass index (BMI), body fat, and total cholesterol in adults with obesity compared with the control in a half year. Daily calorie restriction (CR) is a first-line strategy for adults with obesity to achieve weight loss. However, adherence to CR is difficult to many adults with obesity. Many new strategies have been developed including ADF, time-restricted eating, and a 5:2 diet [2,29,31,32]. ADF is initially used for weight loss. Meanwhile, other benefits, such as improvements in blood pressure, lipid profiles, and insulin sensitivity, have been found in many studies. Many studies have explored the effects of ADF for people with obesity. For example, Varady et al. [27] conducted a study to explore the effects of ADF for coronary artery disease (CAD) risk indicators and body weight in people with obesity, and the results showed that an 8-week ADF intervention could result in a mean weight loss of 5.8% and decrease some several key biomarkers for CAD risk, such as LDL cholesterol, total cholesterol (TC), triacylglycerols, heart rate, and systolic blood pressure (SBP). Hooshiar et al. [33] performed an RCT to investigate the effects of modified alternate-day fasting (MADF) and calorie restriction (CR) on body weight, sleep quality, and daytime sleepiness. They found that ADMF could achieve a greater decrease in weight compared with CR, and MADF might be a beneficial diet for controlling BMI and body weight. (2) **ADF for several different population groups.** In the top 20 highly cited references, seven explored the effects of ADF for non-obese subjects and patients with chronic diseases including diabetes and asthma [9,11,15,17,23,27,28], and insulin resistance occurred in the blue cluster. In addition to obesity, many studies explored the effect of ADF for different populations including healthy, non-obese adults, as well as patients with diabetes and NAFLD. **For healthy, non-obese humans,** ADF is safe, and can increase polyunsaturated free fatty acids (PUFAs), improve the cardiovascular parameters and fat-to-lean ratio, decrease the body weight by 4.5%, and periodically deplete amino acids [9]. **For patients with diabetes,** a randomized controlled trial showed that ADF was effective for glycemic control, while also significantly decreasing BMI, serum triglyceride, body weight, and fat mass [34]. **Two studies investigated the effects of ADF for patients with NAFLD** [8,35]. Johari et al. compared the effects of modified alternate-day calorie restriction (MACR) and normal habitual diet for patients with NAFLD, and they found that ADF was an effective intervention for improving NAFLD-related biomarkers including BMI, weight, and liver transaminases, with a good adherence rate [35]. Recently, a study was conducted to compare the effects of ADF plus exercise to control, exercise alone, and fasting alone on intrahepatic triglyceride (IHTG) content in patients with NAFLD. The results demonstrated that ADF alone was as effective as ADF plus exercise, and they were all better than exercise alone in increasing insulin sensitivity and body weight, fat mass, waist circumference, body weight, and alanine transaminase (ALT) levels [8]. More studies are needed to explore the effects of ADF for diverse populations. It should be noted that sustained ADF may potentiate doxorubicin cardiotoxicity according to a recent study, indicating that the use of ADF should be taken with care in specific populations such as patients receiving doxorubicin treatment [36,37,38].

There were some limitations in our study. The WoSCC database was used in our study, whereas other databases such as Embase and Pubmed were not used since VOSviewer software cannot analyze and visualize co-citation maps using their data. The overall number of publications in the field of ADF remains relatively small; thus, more studies are urgently needed to extend our knowledge about the effects of ADF for human health, as well as take advantage of this approach to improve human health.

In conclusion, this is the first comprehensive bibliometric analysis related to ADF. The main research hotspots and frontiers were ADF for obesity and cardiometabolic risk, and ADF for diverse populations including healthy adults and patients with diabetes, NAFLD, and cancer. The studies about ADF were limited, and more studies are needed to extend our knowledge about ADF, with the aim of improving human health.

## Figures and Tables

**Table 1:** The top 10 productive authors, institutions and countries based on publications.

Items	Publications					
	Ranking	Country	Number	Citations	C/N	*h*-Index
Country	1	USA	98	5555	56.7	36
	2	China	23	579	25.2	10
	3	England	14	313	22.4	10
	4	Australia	10	376	37.6	8
	5	Italy	8	226	28.3	7
	6	Brazil	7	52	7.4	4
	7	Canada	7	200	28.6	5
	8	Iran	6	84	14	5
	9	Switzerland	6	301	50.2	4
	10	Austria	5	216	43.2	4
Institution	1	University of Illinois Chicago	37	2212	59.8	23
	2	National Institutes of Health	10	1439	143.9	8
	3	University of California System	9	490	54.4	9
	4	Louisiana State University System	8	1229	153.6	7
	5	Pennington Biomedical Research Center	7	815	116.4	6
	6	Cornell University	5	117	23.4	4
	7	Medical University of Graz	5	216	43.2	4
	8	University of Michigan	5	33	6.6	3
	9	University of Sydney	5	219	43.8	5
	10	Biotechmed Graz	4	216	54	4
Author	1	Krista A. Varady	43	2628	61.1	25
	2	Cynthia M. Kroeger	17	1337	78.6	15
	3	Monica C. Klempel	13	1314	101.1	12
	4	Kelsey Gabel	13	541	41.6	7
	5	John F. Trepanowski	12	1142	95.2	11
	6	Sofia Cienfuegos	12	191	15.9	6
	7	Kristin Hoddy	11	1041	92.2	10
	8	Mark Ezpeleta	10	123	12.3	6
	9	Surabhi Bhutani	9	939	104.3	9
	10	Faiza Kalam	8	115	14.4	6

The average article citation (C/N) = citations/numbers.

**Table 2:** The top 10 most productive journals.

Ranking	Journal Name	Country	Counts	Citation
1	*Nutrients*	Switzerland	18	318
2	*American Journal of Clinical Nutrition*	USA	6	849
3	*Obesity*	USA	6	528
4	*Journal of Nutritional Biochemistry*	USA	4	84
5	*Mechanisms of Aging and Development*	Switzerland	4	123
6	*Metabolism Clinical and Experimental*	USA	4	217
7	*Nutrition Reviews*	USA	4	203
8	*British Journal of Nutrition*	England	3	65
9	*Cell Metabolism*	USA	3	196
10	*Faseb Journal*	USA	3	31

**Table 3:** The top 20 most highly cited references.

Rank	Title	Journal	Total Citations	Year	First Author
1	Intermittent fasting dissociates beneficial effects of dietary restriction on glucose metabolism and neuronal resistance to injury from calorie intake	*PNAS*	486	2003	R. Michael Anson [10]
2	Alternate-day calorie restriction improves clinical findings and reduces markers of oxidative stress and inflammation in overweight adults with moderate asthma	*Free Radical Biology and Medicine*	414	2007	James B. Johnson [17]
3	Effect of alternate-day fasting on weight loss, weight maintenance, and cardioprotection among metabolically healthy obese adults: A randomized clinical trial	*Jama Internal Medicine*	312	2017	John F. Trepanowski [24]
4	Alternate-day fasting and chronic disease prevention: A review of human and animal trials	*American Journal of Clinical Nutrition*	242	2007	Krista A. Varady [28]
5	Alternate-day fasting in nonobese subjects: Effects on body weight, body composition, and energy metabolism	*American Journal of Clinical Nutrition*	235	2005	Leonie K. Heilbronn [14]
6	Alternate-day fasting for weight loss in normal weight and overweight subjects: a randomized controlled trial	*Nutrition Journal*	234	2013	Krista A. Varady [27]
7	Short-term modified alternate-day fasting: a novel dietary strategy for weight loss and cardioprotection in obese adults	*American Journal of Clinical Nutrition*	223	2009	Krista A. Varady [26]
8	Alternate-day fasting improves physiological and molecular markers of aging in healthy, non-obese humans	*Cell Metabolism*	186	2019	Slaven Stekovic [9]
9	Effects of intermittent fasting on body composition and clinical health markers in humans	*Nutrition Reviews*	170	2015	Grant M. Tinsley [22]
10	Alternate-day fasting and endurance exercise combine to reduce body weight and favorably alter plasma lipids in obese humans	*Obesity*	170	2013	Surabhi Bhutani [12]
11	A randomized pilot study comparing zero-calorie alternate-day fasting to daily caloric restriction in adults with obesity	*Obesity*	169	2016	Victoria A. Catenacci [13]
12	Intermittent fasting vs. daily calorie restriction for type 2 diabetes prevention: A review of human findings	*Translational Research*	164	2014	Adrienne R. Barnosky [11]
13	Intermittent versus daily calorie restriction: Which diet regimen is more effective for weight loss?	*Obesity Reviews*	153	2011	Krista A. Varady [25]
14	Impact of caloric and dietary restriction regimens on markers of health and longevity in humans and animals: A summary of available findings	*Nutrition Journal*	138	2011	John F. Trepanowski [23]
15	Effectiveness of intermittent fasting and time-restricted feeding compared to continuous energy restriction for weight loss	*Nutrients*	134	2019	Corey A. Rynders [20]
16	Health effects of intermittent fasting: Hormesis or harm? A systematic review	*American Journal of Clinical Nutrition*	122	2015	Benjamin D. Horne [16]
17	Glucose tolerance and skeletal muscle gene expression in response to alternate-day fasting	*Obesity Research*	122	2005	Leonie K. Heilbronn [14]
18	Do intermittent diets provide physiological benefits over continuous diets for weight loss? A systematic review of clinical trials	*Molecular and Cellular Endocrinology*	119	2015	Radhika V. Seimon [21]
19	Alternate-day fasting (ADF) with a high-fat diet produces similar weight loss and cardioprotection as ADF with a low-fat diet	*Metabolism—Clinical and Experimental*	107	2013	Monica C. Klempel [19]
20	Fasting for weight loss: An effective strategy or latest dieting trend?	*International Journal of Obesity*	96	2015	Alexandra Johnstone [18]

## Data Availability

The data are available from the corresponding authors upon reasonable request.
